# Heart rate variability in chronic low back pain patients randomized to yoga or standard care

**DOI:** 10.1186/s12906-016-1271-1

**Published:** 2016-08-11

**Authors:** Shirley Telles, Sachin Kumar Sharma, Ram Kumar Gupta, Abhishek Kumar Bhardwaj, Acharya Balkrishna

**Affiliations:** Patanjali Research Foundation, Patanjali Yogpeeth, Haridwar, Uttarakhand 249402 India

**Keywords:** Heart rate variability, Rate of respiration, Yoga, Standard care, Chronic low back pain

## Abstract

**Background:**

Chronic pain can alter the autonomic balance with increased sympathetic activity reflected in altered heart rate variability (HRV). It has been proposed that yoga can be useful to correct the autonomic imbalance in patients with chronic pain who have reduced HRV.

**Methods and designs:**

In the present randomized controlled trial 62 patients with chronic low back pain associated with altered alignment of intervertebral discs (aged between 20 and 45 years, 32 males) were randomized to 2 groups. One group received yoga for 3 months while the other group carried out standard medical care based on the physician's advice. The duration was the same, i.e., 3 months. The heart rate variability and rate of respiration were assessed at baseline and at the end of 3 months.

**Results:**

There was a significant difference in the baseline (pre) values between groups (*p* = 0.008) for respiration rate which was higher in the yoga group. The changes reported below are pre-post comparisons within each group. The yoga group showed a significant (*p* < 0.05; repeated measures ANOVA, *post-hoc* analyses) decrease in the LF power of HRV, rate of respiration and a significant increase in the HF power of HRV and in the pNN50.

**Conclusion:**

The results suggest that yoga practice can shift the autonomic balance towards vagal dominance in patients with chronic low back pain associated with altered alignment of intervertebral discs.

**Trial registration:**

The study is registered with the Clinical Trials Registry of India (CTRI/2012/11/003094) and can be accessed at.

## Background

Non specific chronic low back pain (nCLBP) is prevalent among adults and often leads to functional limitations, psychological symptoms, lower quality of life, and expenditure on health care [1]. Chronic pain is an emotionally and physically debilitating form of pain which activates the body’s stress response and with time can result in a decrease in heart rate variability (HRV) power [2].

The function of the sympathetic and parasympathetic nervous systems is involved in pain regulation [3]. It is also recognized that the changes in autonomic balance which are associated with chronic pain can be assessed by HRV. A systematic meta-analysis reviewed the literature on HRV in different conditions with chronic pain [3]. From a wide range of chronic pain conditions, results from the meta-analysis reflected a consistent, decrease in high frequency (HF) power of HRV in chronic pain suggesting a decrease in parasympathetic activation.

Yoga practice including yoga postures [4], yoga breathing [5] and meditation [6] have been shown to influence the HRV. A systematic review which searched nine databases from their inception up to June 2014, critically assessed the effect of yoga on HRV [7]. Ten trials reported favorable effects of yoga on different measures of the HRV. However the results did not provide convincing evidence that yoga can modulate the HRV in either patients or healthy persons, which was believed to be due to multiple methodological weaknesses in the research methods of the studies reviewed. Despite this report currently it cannot be said that yoga does not influence the HRV; instead the effect of yoga on the HRV requires thorough investigation. To our knowledge none of these studies assessed the HRV in patients with chronic low back pain who practiced yoga. This is unique to the present study.

With this background the present study was planned to assess the effect of yoga on the HRV in patients with chronic low back pain associated with altered alignment of intervertebral discs. In the present study to assess the function of the autonomic nervous system the energy in the HRV series for two specific bands was studied, viz. the low frequency band (0.05–0.15 Hz) and high frequency band (0.15–0.50 Hz). The values of the low frequency (LF) and high frequency (HF) band were expressed as normalized units. The LF/HF ratio was also calculated. In addition to this the following components of time domain analysis of HRV were obtained: (i) the mean RR interval (the mean of the intervals between adjacent QRS complexes or the instantaneous heart rate), (ii) RMSSD (the square root of the mean of the sum of the squares of differences between adjacent NN intervals), (iii) NN50 (the number of interval differences of successive NN intervals greater than 50ms), and (iv) pNN50 (the proportion derived by dividing NN50 by the total number of NN intervals).

Concurrent monitoring of the HRV and respiration is emphasized as quiet respiration is associated with simultaneous phasic change in the activity of medullary vagal cardiac motonuclei [8]. Hence the rate of respiration was also monitored.

Among non-pharmacological interventions yoga has been shown to be useful in reducing self reported pain and improving the level of functioning in patients with low back pain [1, 9]. A recent randomized controlled trial [9] showed that 3 months of yoga practice significantly decreased self reported chronic back pain in patients with altered alignment of intervertebral disc changes [CTRI/2012/11/003094]. In the study 126 patients were included. The HRV and respiration (which take approximately 15 min to record per patient) were intended to assess in a randomly selected sub-set of 62 of the 126 patients, who belonged to both groups and the findings are presented here. The focus and aim of the present study was different from the study on the larger group of 126 patients [CTRI/2012/11/003094], i.e., it was to assess the autonomic balance based on the HRV in patients with chronic low back pain following yoga as a therapeutic intervention.

## Methods

### Study design

**Fig. 1:**
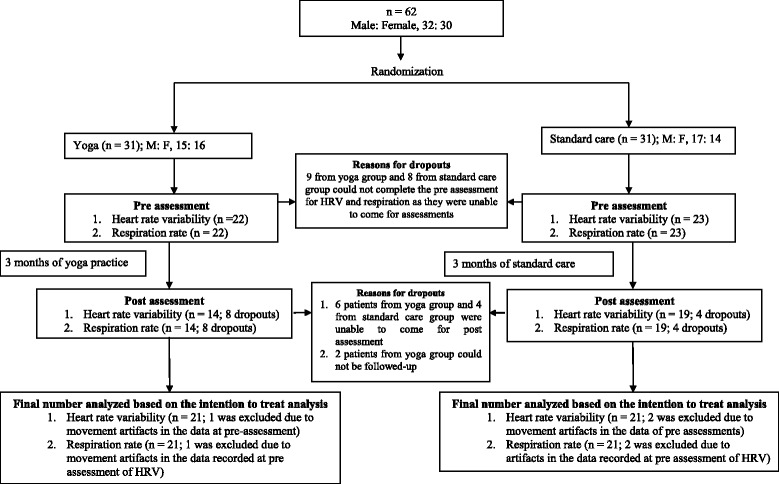
Trial profile of the study

The present study was a sub-analysis of a 2-arm parallel group randomized controlled trial [CTRI/2012/11/003094] carried out between May to July 2011 and the follow-up was done between October to December 2011. In the present study the patients were randomized as two groups, (i) a yoga group and (ii) a standard care group using a computer generated random number table by a staff member who had no other involvement in the trial. The randomization was carried out as follows. (i) The participants were given numbers from 1 to 62 which were not dependent on their name, or the order in which they were enrolled. (ii) Sixty two random numbers were generated. (iii) These 62 random numbers were written in a separate column adjacent to the serial number of the participants. (iv) The 62 random numbers were written on identical pieces of paper. (v) After this they were folded identically, manually mixed, and the slips of paper were then placed alternately in two boxes labeled A and B by a person who was unaware about the details of the trial. (vi) Individuals allotted to Box A were given yoga intervention while individuals allotted to Box B were assigned to standard care. The allocation of the patients to the two groups was 1:1 ratio. The patients were assessed at baseline and after 3 months. Figure 1 shows the number of patients at different stages of the trial and final number used for analysis. The signed informed consent from each patient was taken after explaining the study design to each of them. The study was approved by the ethics committee of Patanjali Research Foundation and is registered with the Clinical Trials Registry of India (CTRI/2012/11/003094). The study is reported according to standard CONSORT guidelines.

### Patients

Sixty-two patients with chronic low back pain associated with altered alignment of intervertebral discs, of both sexes (32 male), ages between 20 and 45 years with group average age ± S.D.; 36.2 ± 6.4 years were enrolled for the study. Seventeen patients could not complete the initial assessments of the HRV and respiration as they could not spare the 15 min required for testing. Therefore 45 patients (23 males) with an age range between 20 and 45 years, with group average age ± S.D.; 35.6 ± 6.2 years were included in the present study. The yoga group comprised of 22 patients (10 males) with group average age ± S.D.; 34.6 ± 6.5 years while there were 23 patients (13 males) in the standard care group with group average age ± S.D.; 36.6 ± 6.0 years. Estimation of sample size was not done prior to the study. However the *post-hoc* analyses showed that for the present study, with the sample size as 21 (the final number used for analysis on intention-to-treat basis) in each group, and with the Cohen’s *d* of 0.53 for the yoga group obtained from the mean and S.D. of the LF component of HRV, the power was 0.9958 [10], while the level of significance (α) was 0.05. With the SPSS software Bonferroni adjustment multiplies the uncorrected *p*-value by the number of comparisons; hence α remains unchanged. Recruitment was done by distributing leaflets in a local hospital and advertising in the local newspaper. The study was conducted in a yoga and ayurveda center located in the north of India. No incentive was given to the patients to enroll in the study. Patients were included in the trial if (i) their ages were between 20 and 45 years and (ii) they had herniation of at least 1 disc based on the MRI report. Patients were excluded from the trial if they had (i) spinal surgery, (ii) osteoporosis, (iii) pregnancy, (iv) severe cardiovascular or metabolic diseases, and (v) extra-systoles, which would influence the HRV reading. Exclusion criteria also included cardiac pacemakers and metal implants which would not allow MRI recordings to be done. Two patients who had tethered spinal cord were excluded from the trial.

### Interventions

#### Yoga intervention

The yoga group was taught the following components of yoga practice: (i) specific yoga postures (*asanas*), (ii) breathing techniques (*pranayamas*), and (iii) relaxation techniques, from an experienced yoga instructor. These components were modified by an orthopedic surgeon, particularly for patients with back pain [11]. The details of yoga practice are given in Table 1. The yoga group first attended a course which consisted of three group classes per week for 2 weeks in order to develop a home practice, which they were asked to continue daily for the rest of the trial. Weekly yoga classes were available throughout the trial. Yoga was taught by a trained instructor who had a 2 year post-graduate degree in yoga (the training included both theory and practice). Yoga class attendance was monitored by another person.

**Table 1:** Details of the yoga intervention

Sl. no.	Type of practice	Duration
1	Meditation on the *Sanskrit* syllable, OM	5 min
2	Relaxation *(* in *Shavasana)*	5 min
3	Light exercises and specific yoga postures (*asanas*)	25 min
	• Hold and rotate knees in circles (both directions)	
	• Roll onto one side	
	• Raise one leg at a time	
	• Lift the head and shoulders	
	• Pelvic tilting	
	• Touch the face to flexed knees (with one leg)	
	• Supine twist (*Supta Matsyendrasana*)	
	• Cobra pose (*Bhujangasana*)	
	• Locust pose (*Shalabhasana*)	
	• Child pose (*Balasana*)	
4	Guided relaxation	10 min
	• *Shavasana*/yoganidra	
5	*Pranayama*	
	• Slow breathing	7 min
	• *Kapalabhati* (1 stroke per second, not forcefully)	3 min
6	Meditation and concluding prayer	5 min
	Total duration	60 min

#### Standard care

The standard care group was asked to carry on with the routine medical care suggested by their physician consisting of (i) analgesics and (ii) non-steroidal anti-inflammatory medication. The standard care group was also given advice about which activities would worsen their condition and asked to avoid them.

### Assessments

#### EKG and Respiratory variables

Assessment of EKG and rate of respiration were conducted between 10:30 am to 12:30 pm and 3:30 pm to 5:30 pm in a sound attenuated and air-conditioned laboratory where patients were assessed while seated with back support. Repeat assessments were carried out at the same time of the day. Patients were asked to abstain from alcohol and/or any caffeinated beverages within 24 h of the assessment. They were instructed to breathe at their normal rate. The EKG and rate of respiration were obtained using a MP45 data acquisition system (BIOPAC System Inc, U.S.A). The EKG was recorded using Ag/AgCl pre-gelled electrodes (Tyco Healthcare, Germany) and a standard bipolar Limb Lead II configuration. The EKG data were acquired at the sampling rate of 1024 Hz. The respiration was recorded using a respiratory stethograph transducer fixed around the trunk about 8 cm below the lower costal margin when the patients sat erect.

### Data extraction

The HRV and respiration rate was recorded for 5 min at baseline and after 3 months of the intervention. HRV was inspected off-line and only noise free data were included. One patient from the yoga group and two patients from the standard care group were excluded due to artifacts in the HRV data. Fast Fourier Transform analysis (FFT) of R-R interval series was done to obtain the spectrum of HRV using software developed by the Biosignal Analysis and Medical Imaging Group, University of Eastern Finland, Kubios HRV. The frequency domain and time domain analysis of HRV was done separately. The assessments (HRV and respiration rate) were blind scored by an investigator who was unaware of the group to which a participant belonged.

### Statistical methods

Repeated measures analysis of variance (RM-ANOVA) followed by Bonferroni adjusted *post-hoc* analyses were carried out using PASW Version 18.0. There was one Within subjects factor i.e., States, with two levels (pre and post) and one Between subjects factor i.e., Groups with two levels (yoga and standard care). Missing value analysis was performed. Statistical significance (α) was set at 0.05 (Bonferroni adjusted). Generally the Bonferroni adjustment is calculated by dividing the level of significance by the number of comparisons, but SPSS software uses a mathematically equivalent adjustment by taking the uncorrected *p*-values and multiplying them by the number of comparisons made. Hence α remains unchanged. The trial profile is given in Fig. 1.

## Results

### Repeated Measures Analysis of Variance (ANOVA)

#### Time domain analysis

There was a significant interaction between States X Groups in (i) RMSSD with *F* = 8.433, *df* = 1,40, *p* = 0.006 and (ii) pNN50 with *F* = 4.382, *df* = 1,40, *p* = 0.043.

#### Frequency domain analysis

There were no significant difference between States, Groups or their interaction for LF, HF, and LF/HF.

#### Respiration rate

The respiration rate showed a significant difference between the (i) States with *F* = 10.963, *df* = 1,40.0, *p* = 0.002, and (ii) Groups with *F* = 8.835, *df* = 1,40.0, *p* = 0.005.

### Post-hoc analyses

There was a significant difference in the pre values between groups (*p* = 0.008) for respiration rate which was higher in the yoga group with 95 % CI of [2.986, 0.482]. The other differences mentioned below were pre-post comparisons within the group.

#### Time domain analysis

The yoga group showed a significant increase in pNN50 (*p* = 0.032) with 95 % CI of [-0.507, -10.807].

#### Frequency domain analysis

The yoga group showed a (i) significant decrease in LF power of the HRV (*p* = 0.049) with 95 % CI of [13.858, 0.047] and (ii) a significant increase in the HF power of the HRV (*p* = 0.049) with 95 % CI of [-0.026, -13.851].

#### Respiration rate

In a pre post comparison the yoga group showed a significant decrease in respiration rate (*p* = 0.001) with 95 % CI of [2.448, 0.731].

The group mean values ± S.D. for frequency domain analysis and time domain analysis of HRV along with respiration rate are given in Table 2. ANOVA values for frequency domain analysis and time domain analysis of the HRV along with respiration rate are given in Table 3. None of the patients reported any adverse event during the intervention which was specifically checked during the interaction with them.

**Table 2:** Frequency domain analysis and time domain analysis of the of the heart rate variability and respiration rate (RR) based on intention-to-treat analysis

Variables	Yoga (*n* = 21)			Standard care (*n* = 21)		
	Pre	Post	Cohen’s *d*	Pre	Post	Cohen’s *d*
LF (n.u)	57.8 ± 14.2	50.8 ± 12.0*	0.53	56.7 ± 15.5	58.7 ± 16.9	0.12
HF (n.u)	42.2 ± 14.2	49.2 ± 12.0*	0.53	43.3 ± 15.5	41.3 ± 16.9	0.12
LF/HF	1.71 ± 1.24	1.15 ± .53	0.63	1.68 ± 1.15	1.94 ± 1.53	0.65
Mean RR	0.76 ± 0.09	0.79 ± 0.10	0.32	0.76 ± 0.08	0.75 ± 0.08	0.13
RMSSD	56.47 ± 49.44	79.14 ± 50.68	0.45	62.54 ± 55.52	32.37 ± 20.06	0.80
NN50	28.14 ± 39.13	43.88 ± 38.41	0.41	29.05 ± 37.02	21.14 ± 33.36	0.22
pNN50	7.75 ± 11.64	13.40 ± 13.27*	0.45	7.65 ± 10.22	5.77 ± 9.06	0.20
Respiration rate (CPM)	13.01 ± 2.60†	11.42 ± .98**	0.89	11.29 ± 1.12	10.88 ± .97	0.39

Values are group mean ± SD

*p < 0.05; **p < 0.01, *post-hoc* analyses with Bonferroni adjustment, post values compared with pre values

†*p* < 0.01, *post-hoc* analyses with Bonferroni adjustment, pre value of yoga compared with pre value of standard care

**Table 3:** ANOVA table for the frequency domain analysis and time domain analysis of heart rate variability and respiration rate

SI. no.	Factors	Variable	*F*	df	Huynh-Feldt ε	*p* value
I	Within Subjects	LF	1.079	1,40	1	0.305
		HF	1.071	1,40	1	0.307
		LF/HF	0.511	1,40	1	0.479
		Mean RR	0.277	1,40	1	0.602
		RMSSD	0.170	1,40	1	0.682
		NN50	0.382	1,40	1	0.540
		pNN50	1.095	1,40	1	0.302
		Respiration rate (CPM)	10.963	1,40	1	0.002
II	Between Subjects	LF	0.770	1,40	1	0.386
		HF	0.766	1,40	1	0.387
		LF/HF	1.687	1,40	1	0.202
		Mean RR	0.894	1,40	1	0.350
		RMSSD	3.460	1,40	1	0.070
		NN50	1.316	1,40	1	0.258
		pNN50	1.735	1,40	1	0.195
		Respiration rate (CPM)	8.835	1,40	1	0.005
III	Groups × States	LF	3.381	1,40 (Groups) × 40 (States)	1	0.073
		HF	3.364	1,40 (Groups) × 40 (States)	1	0.074
		LF/HF	3.955	1,40 (Groups) × 40 (States)	1	0.054
		Mean RR	1.763	1,40 (Groups) × 40 (States)	1	0.192
		RMSSD	8.433	1,40 (Groups) × 40 (States)	1	0.006
		NN50	3.478	1,40 (Groups) × 40 (States)	1	0.070
		pNN50	4.382	1,40 (Groups) × 40 (States)	1	0.043
		Respiration rate (CPM)	3.922	1,40 (Groups) × 40 (States)	1	0.055

## Discussion

Patients with chronic low back pain (LBP) showed an increase in pNN50 (time domain analysis) and in the HF power (frequency domain analysis) with a decrease in LF power (frequency domain analysis) of the heart rate variability (HRV), after 3 months of yoga compared to before. The single between groups difference was a higher baseline respiration rate in the yoga group.

The increase in pNN50, increase in HF power and decrease in LF power of HRV, in patients with LBP after 3 months of yoga practice are suggestive of a shift in autonomic balance. The function of the autonomic nervous system is involved in pain regulation [3]. One of the methods to assess autonomic balance is the HRV. A systematic meta-analysis which followed the standard guidelines for systematic reviews and meta-analyses critically reviewed the literature on HRV in conditions associated with chronic pain [3]. Fifty-one studies, out of 17,350 fulfilled the inclusion criteria. Across a wide range of conditions pooled results from the meta-analysis reflected a consistent, moderate-large decrease in HF power of the HRV in chronic pain suggesting a decrease in parasympathetic activity. Specific yoga practices including yoga breathing and certain meditation techniques also influence the HRV with a shift towards greater parasympathetic activity [4–6]. However different results were obtained from a systematic review which searched 9 databases from their inception till June 2014, to critically assess the effects of yoga on the HRV [7]. Fourteen trials out of 1966 searched results met the inclusion criteria. Ten trials reported favorable effects of yoga on different measures of the HRV. However the results did not provide convincing evidence based on the Cochrane collaboration’s tool that yoga can modulate the HRV in either patients or healthy persons. This was believed to be due to several methodological weaknesses of the research methods in the studies reviewed. Despite this review at present it cannot be said conclusively that yoga has no effect on the HRV. It would be more correct to say that the effect of yoga on the HRV requires further investigation with rigorous research methods. With the speculation that yoga influences the HRV and that HRV is modified by chronic pain the present study was designed to measure the HRV in patients with chronic low back pain associated with altered alignment of intervertebral discs.

The respiration was simultaneously monitored as it has been demonstrated that the HRV and respiration have a connection [12, 13]. The decrease in the respiration following yoga (within session comparison) could be related to various physiological and psychological factors [14].

The present results resemble the findings of an earlier study carried out on healthy male volunteers [15]. In the earlier study [15] eleven experienced Iyengar yoga practitioners had five training units each of 90 min, once a week over 5 successive weeks. Two sessions were of Iyengar yoga while in three sessions they practiced resting and walking (called the placebo). The yoga program included specific yoga postures (*asanas*) including supine relaxation (*shavasana*). All the participants underwent a 24 h Holter monitoring on each training day and measures of HRV were determined hourly by a blinded observer. The study reported that the measures of HRV i.e., SDNNi (mean standard deviation of normal beat to beat of the EKG intervals for all 5 min intervals), RMSSD (the square root of the mean of the sum of squares of differences between adjacent NN intervals) and mean RR interval which are strongly associated with vagal tone were significantly higher on the days of yoga intervention when compared to the placebo intervention and the control group.

Since the present study resembles the findings of the earlier study cited above [15] it would be ideal to compare the results of the present study with the earlier study. In the earlier study [15] measures of HRV (SDNNi, RMSSD and mean RR) which are strongly related to vagal tone were significantly higher on the days of yoga intervention. In the present study there was a significant increase in pNN50 which is also a marker of increased vagal tone following yoga. The earlier study [15] reported changes in normal individuals on the days of practice of yoga postures. The present study was a longitudinal prospective trial in patients with chronic low back pain in which the HRV was assessed at baseline and after 3 months. However the yoga practice did include yoga postures as did the earlier study [15]. The earlier study [15] did not report the frequency domain analysis of HRV i.e., LF, HF and LF/HF. Despite these differences both studies (the earlier study [15] and the present study) showed significant changes in the HRV that are strongly associated with vagal modulation.

A systematic meta-analysis [16] of 27 controlled trials in which patients with ischemic heart disease were taught different relaxation techniques such as progressive muscle relaxation, biofeedback, breath relaxation and psychological training among other practices, reported that intense supervised relaxation practice enhances recovery from an ischemic cardiac event. Out of twenty-seven controlled studies that were included in the meta-analysis, 3 reported a significant increase in the HRV following relaxation techniques [17–19]. The yoga intervention taught to the patients of the present study is a combination of relaxation techniques and psychological training. For example, in the present study stretching and relaxation of the muscles (during the practice of light exercise and specific postures), slow breathing and relaxation in the supine posture (*shavasana*) and meditation can be compared with several of the mind-body practices assessed in the meta-analysis.

### Limitations

The main limitation of the present study was that there was no attempt to assess other measures of autonomic activity other than the time and frequency domain analysis of HRV. Also, due to time constraints HRV recording was carried out for 5 min, which is the minimum duration specified by the task Force of the European Society of Cardiology and the North American Society of Pacing and Electrophysiology [20]. While all attempts were made to ensure that patients were relaxed during the recording, the short duration (5 min) is a limitation of the study. Overall, the present results indicate that patients with chronic low back pain associated with altered alignment of intervertebral discs, show a shift towards vagal dominance after 3 months of yoga. Hence potential autonomic dysfunction associated with the chronic pain [3] may be prevented or corrected.

## Conclusions

The present study was conducted on patients with chronic low back pain associated with altered alignment of intervertebral discs. Chronic pain is usually associated with increased sympathetic activity. In the present trial after 3 months of yoga the patients with chronic low back pain associated with altered alignment of intervertebral discs showed heart rate variability changes suggesting an increase in vagal activity compared to baseline. The control group showed no change.

## Abbreviations

ANOVA, Analysis of Variance; CI, Confidence Interval; CONSORT, Consolidated Standards of Reporting Trials; CTRI, Clinical Trials Registry of India; EKG, Electrocardiogram; FFT, Fast Fourier Transform; HF, high frequency; HRV, heart rate variability; LBP, low back pain; LF, low frequency; MRI, Magnetic Resonance Imaging; nCLBP, Non specific chronic low back pain; NN50, the number of interval differences of successive NN intervals greater than 50ms; PASW, Predictive Analytics SoftWare; pNN50, the proportion derived by dividing NN50 by the total number of NN intervals; RMSSD, the square root of the mean of the sum of the squares of differences between adjacent NN intervals; RR interval, the mean of the intervals between adjacent QRS complexes or the instantaneous heart rate; S.D, Standard deviation; SDNNi, standard deviation of NN intervals for 5-minute segments; SPSS, Statistical Package for the Social Sciences
